# Age‐Dependent Correlation of Pulmonary Function Impairment and Aortic Arch Stiffness in Mice

**DOI:** 10.1002/pdi3.2525

**Published:** 2025-04-02

**Authors:** Ayman Isbatan, Samuel M. Lee, Ali Imran Sarwar, Yuxing Yuan, Sunny Chen, Maricela Castellon, Dan A. Marian, Richard D. Minshall, Jie Tian, Irena Levitan, Jiwang Chen

**Affiliations:** ^1^ Cardiovascular Research Center University of Illinois at Chicago Chicago Illinois USA; ^2^ Department of Cardiology, National Clinical Research Center for Child Health and Disorders, China International Science and Technology Cooperation Base of Child Development and Critical Disorders, Key Laboratory of Children's Important Organ Development and Diseases of Chongqing Municipal Health Commission, National Clinical Key Cardiovascular Speciality Children’s Hospital of Chongqing Medical University Chongqing China; ^3^ Department of Anesthesiology University of Illinois at Chicago Chicago Illinois USA; ^4^ Department of Pharmacology University of Illinois at Chicago Chicago Illinois USA; ^5^ Department of Medicine University of Illinois at Chicago Chicago Illinois USA

**Keywords:** aging, aortic arch stiffness, echocardiography, mouse model, pulmonary function

## Abstract

Progressive aging is known to negatively affect cardiopulmonary function, which increases the risk of developing cardiac/respiratory diseases. However, the relationship between the decline in pulmonary function and aortic arch stiffness in aging is not well understood. This study investigated the correlation between lung function impairment and aortic arch stiffness in male and female C57BL/6 mice from 2 to 52 weeks of age. Lung function was assessed using forced oscillation ventilation and aortic arch stiffness was measured through echocardiography. Our results show a progressive decline in lung function markers such as respiratory system resistance (Rrs) and tissue elastance (H) with age, alongside an increase in inspiratory capacity (IC) and compliance of the respiratory system (Crs). Aortic arch stiffness significantly increased with age, particularly during early adulthood, and was found to be a strong predictor of lung function impairment in both sexes. While linear regression models indicated that body weight was a more accurate predictor of lung function variability, aortic arch stiffness emerged as a reliable marker for the decline in pulmonary function. These findings suggest that aortic arch stiffness can serve as an early indicator of declining lung function and therefore provide a noninvasive method to assess cardiopulmonary health in aging populations. Future studies should explore the molecular mechanisms underlying these changes and extend the investigation to older mice to fully understand the long‐term impacts of aging on cardiopulmonary function.

## Graphical abstract

This study examined the relationship between aortic arch stiffness and lung function in aging mice. Multiple linear regression modeling showed moderate to strong correlations between the rise in aortic arch stiffness and changes in lung function as the mice progressively aged.

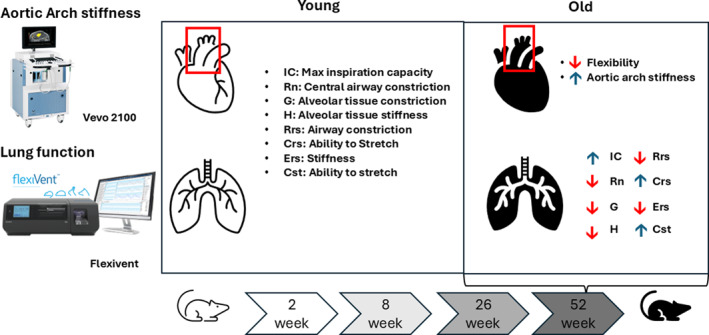

## 1 Introduction

The effect of senescence on lung dysfunction is a well‐established phenomenon characterized by structural and functional changes in both the respiratory system and chest wall [1]. As humans age, lung compliance, the ability of the lungs to stretch and expand in response to breathing, increases [2]. The progressive rise in lung compliance due to aging can be detrimental and can lead to reduced elastic recoil, decreased respiratory drive, and compromised O_2_/CO_2_ exchange [3]. As a consequence, the risk of developing respiratory diseases, such as chronic obstructive pulmonary disease (COPD) or emphysema, progressively rises as we age [4]. To adjust for compromised lung function, cardiac workload is increased as the heart compensates for the reduced amount of oxygen delivered to the bloodstream. Although clinical studies have examined the impact of aging on lung function and heart failure, factors such as genetics, lifestyle, and environmental factors, can limit our ability to interpret these findings in the context of aging. Furthermore, because elderly populations are more susceptible to the development of acute and chronic pulmonary diseases, it can be difficult to assess the impact of aging alone in the clinical setting [2, 5].

Despite their differences, mice are a convenient animal model to assess aging and its impact on cardiopulmonary function due to their rapid developmental cycle. Although studies have shown that lung function declines as mice age, the relationship between the effects of aging and cardiopulmonary function requires further investigation. Aortic arch stiffness, a measure of blood vessel wall elasticity in the aorta, is used as an aging marker [6] and a reliable noninvasive predictor for early cardiovascular diseases [7]. The loss of elasticity in the ascending and descending aortic walls can compromise hemodynamic function in the heart as the aortic arch's ability to expand and contract during systole and diastole is reduced [8, 9]. This increases the workload of the heart, leading to the development of cardiac hypertrophy and eventually heart failure. The relationship between lung function and aortic arch stiffness in the context of aging is poorly understood. This study investigated this relationship in a noninvasive manner by using male and female mice, aged 2–52 weeks, and assessing them by forced expiration volume (FEV) and echocardiography.

## 2 Materials & Methods

### 2.1 Mice

C57BL/6 male and female mice were used for this study, which was approved by the Institutional Animal Care and Use Committee of the University of Illinois at Chicago. All experiments were performed in accordance with the guidelines and regulations of the University of Illinois at Chicago. Lung and cardiac functions were assessed at 2, 8, 26, and 52 weeks of age.

### 2.2 Lung Function Measurement

Mice were anesthetized with an intraperitoneal injection of 100 mg/kg ketamine (Hospira, Lake Forest, Illinois, USA) and 10 mg/kg xylazine (Akorn, Lake Forest, Illinois, USA), followed by intubation with a 20G intravenous catheter (BD, Sandy, Utah, USA). The animals were then connected to a flexiVent system (Scireq, Montreal, QC, Canada) and mechanically ventilated at a respiratory rate of 150 breaths/min, a tidal volume of 10 mL/kg, and a PEEP set at 3 cmH_2_O. Forced oscillation measurements were completed using the single‐FOT maneuver (“Snapshot‐150 perturbation”) and the broadband FOT maneuver (“Quick Prime‐3 perturbation”).

The single‐FOT measurements were fitted to a single‐compartment model to deduce respiratory system resistance (Rrs) and compliance of the respiratory system (Crs). The multifrequency FOT measurements were fitted to a constant phase model to acquire Newtonian resistance (Rn), tissue damping (G), and tissue elastance (H). Pressure–volume (PV) loops were also generated to derive the compliance (C) of the respiratory system, an estimate of inspiratory capacity (A), curvature of the upper portion of the deflation limb of the PV curve (K), and the area enclosed by the PV loop (Area).

The negative pressure‐driven forced expiratory (NPFE) maneuver was then performed by inflating the mouse lungs to a pressure of 30 cmH_2_O over 1 s, then holding this pressure for 2 s before connecting the animal's airways to the negative pressure reservoir (−50 cmH_2_O) for 2 s. The forced expired volume over 0.1 s (FEV0.1), forced vital capacity (FVC), and peak expiratory flow (PEF) were calculated directly from the flow‐volume loop produced during lung deflation. In every mouse, each maneuver was repeated until three acceptable measurements were recorded. The median of at least three acceptable measurements was then calculated.

### 2.3 Transthoracic Echocardiography (Left/Right Ventricular Function and Aortic Arch Stiffness)

Mice were anesthetized in an induction chamber with 3% isoflurane. Once complete anesthesia was confirmed, mice were transferred onto an animal stage where anesthesia was maintained by facemask with 1% isoflurane. Heart rate was maintained between 400 and 500 beats per minute while body temperature was kept between 36° and 38°C using a heat lamp and a rectal temperature probe. Once the mouse was secured onto the animal stage, transthoracic echocardiography was performed using a Vevo 2100 (VisualSonics Inc., Toronto, ON, Canada) with an MS550D transducer (22–55 MHz). Images were then measured using VevoLab, version 5.8.1, to assess aortic arch stiffness and left ventricular (LV) and right ventricular (RV) function.

To measure aortic arch stiffness, the protocol reported previously [6] was used to assess aortic arch pulse wave velocity (PWV). From the suprasternal axis, the aortic arch was identified and B‐mode images were captured followed by color Doppler‐imaging to record the peak velocities of the ascending and descending aorta. Using the same image plane, PWV in the aortic arch was then measured. Time (T1) was measured from the onset of the R wave of the QRS complex to the onset of the ascending aortic Doppler waveform specifically at the aortic annulus. For the descending aorta, time (T2) was measured from the onset of the QRS complex to the onset of the descending aortic Doppler waveform as distally as possible along the aortic arch. To reduce variability, T1 and T2 values were both averaged over 10 cardiac cycles. Finally, the aortic arch distance (D1) was measured between the two sample volume positions along the central axis of the aortic arch to determine the PWV which was calculated as: PWV = D1/(T2 − T1) (cm/s).

Left ventricular diastolic function was assessed by the apical four‐chamber view where color flow and pulsed wave Doppler images (M‐mode) were captured at the mitral valves where early filling (E) and late filling (A) wave peak velocities were measured. Tissue Doppler measurements were captured at the left ventricular posterior wall (M‐mode) to capture the E prime (e′) and A prime waves (a′). From the M‐mode images, the color and tissue Doppler measurements were captured over 10 cycles and used to calculate the E/A, E′/A′, and E′/e′ ratios. LV systolic function was acquired in M‐mode echocardiograms from the LV parasternal short‐axis (SAX) view, recorded at the level of the papillary muscles. LV ejection fraction (EF), cardiac output (CO), stroke volume (SV), and fractional shortening (FS) were measured.

Right ventricular free wall thickness (RVFWT) was measured during end‐diastole in the parasternal short‐axis mitral valve level two‐dimensional (2D) or parasternal long‐axis RV outflow tract level M‐mode. Pulsed wave Doppler echo was used to record the pulmonary artery blood outflow at the level of the aortic valve in the short‐axis view to measure pulmonary acceleration time (PAT) and pulmonary ejection time (PET). Tricuspid annular plane systolic excursion (TAPSE) was measured in 2D M‐mode echocardiograms from the apical four‐chamber view by positioning the cursor on the lateral tricuspid annulus near the free RV wall and aligning it as close as possible to the apex of the heart.

### 2.4 Data Analyses

All *t*‐tests, one‐way ANOVA with post hoc analyses, simple/multiple linear regression analyses, and graphs were generated using GraphPad Prism, version 10.2.2 (GraphPad Software, La Jolla, CA, USA). *p* values less than 0.05 were considered statistically significant. All data are presented as mean ± standard error of the mean (SEM).

## 3 Results

### 3.1 The Progressive Decline of Lung Function From Adolescence to Adulthood Is Attributed to the Loss of Elasticity and Chest Wall Compliance

**FIGURE 1:**
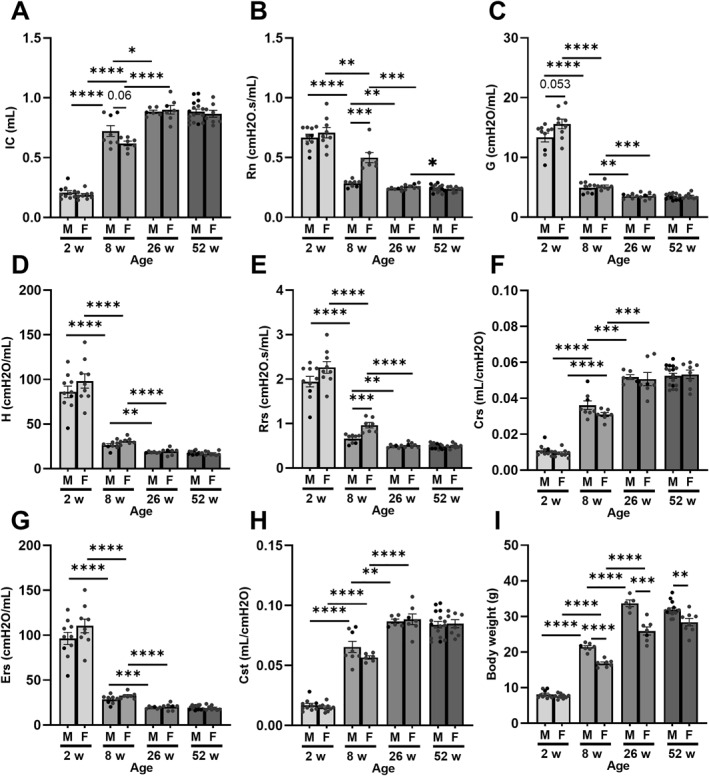
Characterization of lung function in aged male and female mice by flexiVent. (A) Inspiratory capacity (IC), (B) Newtonian resistance (Rn), (C) tissue damping (G), (D) tissue elastance (H), (E) resistance to respiratory system (Rrs), (F) compliance of the respiratory system (Crs), (G) elastance of the respiratory system (Ers), (H) quasi‐static compliance (Cst), and (I) body weight. Sample size was 5–16 per group, data presented as mean ± SEM. Statistical significance presented as * *p* < 0.05, ** *p* < 0.01, *** *p* < 0.001, and **** *p* < 0.0001.

To determine whether there was a decline in lung function, mice were assessed by forced oscillation ventilation at 2, 8, 26, and 52 weeks. From as early as 2 weeks, inspiratory capacity (IC), compliance of the respiratory system (Crs), and quasi‐static compliance (Cst) dramatically increased by 2 months. This rise continued at 6 months of age before plateauing off after a year (Figure 1A,F,H). These findings suggest that as mice age, the maximum volume of air which can be inspired rises progressively due to increased stretch capacity. Inversely, Newtonian resistance (Rn), tissue damping (G), tissue elastance (H), resistance to respiratory system (Rrs), and elastance of the respiratory system (Ers) all declined until 6 months of age (Figure 1B–E,G). Because these are traditional markers of alveolar tissue constriction/stiffness and central air constriction, these results suggest that lungs relax as they progressively mature. Interestingly, the same trends were reported in females with minor differences detected for Rn and Rrs at 8 weeks of age. The decline in Rn (Males‐2 weeks: 0.66 ± 0.029 to 8 weeks: 0.28 ± 0.010 vs. Females‐2 weeks: 0.71 ± 0.043 to 8 weeks: 0.50 ± 0.042) and Rrs (Males‐2 weeks: 1.94 ± 0.12 to 8 weeks: 0.66 ± 0.035 vs. Females‐2 weeks: 2.26 ± 0.13 to 8 weeks: 0.97 ± 0.059) from 2 to 8 weeks was less severe in females compared to males (*p* < 0.001). These findings suggest that gender has a minor but noticeable effect on lung function as mice transition from adolescence to adulthood. Whether the decline in lung elasticity in the alveolar tissue and central airways is influenced by gender requires further investigation.

To determine whether age and body weight were important independent variables for lung function, linear regression models were generated for male and female mice (Figures S1–S4). For all the flexiVent parameters assessed, age and body weight were both important predictors of lung function (*p* < 0.05). However, goodness of fit modeling suggests that body weight is a more accurate predictor for explaining the variability reported in all functional readouts of lung function (*R* ^2^: ∼0.83–0.95) compared to age (*R* ^2^: ∼0.51–0.75). ANCOVA analyses reported similar findings when univariate analyses were performed and showed that both age and weight were positively correlated with IC, Crs, and Cst whereas Rn, G, H, Rrs, and Ers (*p* < 0.001) were negatively correlated (Table S1). However, when multivariate regression analyses were performed and corrected for gender differences, the results showed that body weight was still positively correlated with IC, Crs, and Cst and negatively correlated with Rn, G, H, Rrs, and Ers (*p* < 0.01); however, no significant correlation between age and lung function indices was detected.

### 3.2 Aortic Arch Stiffness Rapidly Increases During Adolescence to Early to Mid Stages of Adulthood

**FIGURE 2:**
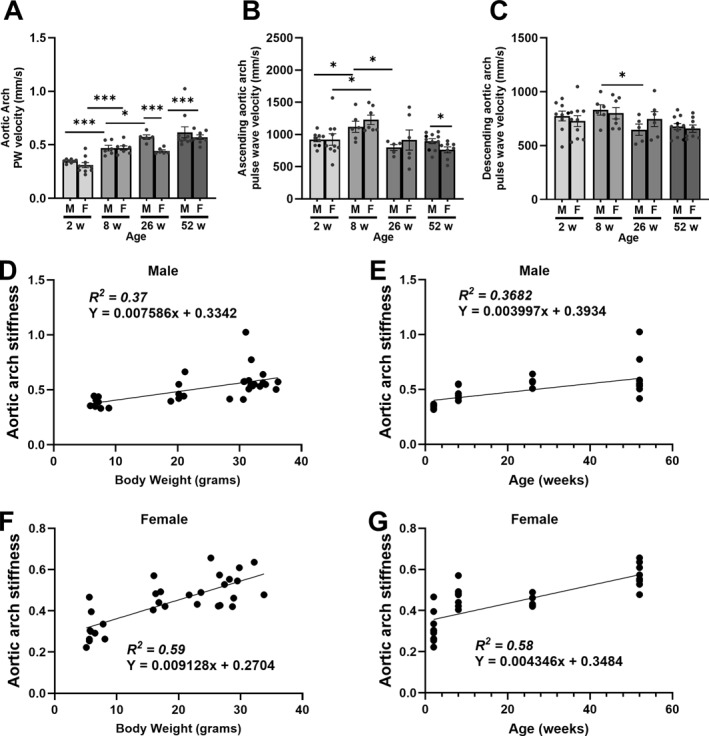
Aortic stiffness measurements of aged male and female mice assessed through echocardiography. (A) Aortic arch stiffness was determined through measurement of the (B) ascending and (C) descending aortic arch pulse wave velocity. Linear regression models of aortic arch stiffness was assessed in male (D) body weight or (E) age and female (F) body weight or (G) age. Sample size was 5–16 per group, data presented as mean ± SEM. Statistical significance presented as * *p* < 0.05 and *** *p* < 0.001.

To determine the relationship between aortic arc stiffness and its relationship with age, color Doppler measurements of the ascending and descending aorta were assessed. Our results revealed that aortic arch stiffness gradually increased in both male and female mice as they grew older (Figure 2). By 4 weeks of age, aortic arch stiffness significantly increased (2‐weeks: 0.35 ± 0.007 vs. 2‐months: 0.47 ± 0.026, *p*‐value = 0.0002) during adolescence before plateauing off after 6 months (Male‐26 weeks: 0.51 ± 0.057 vs. 52 weeks: 0.62 ± 0.051) in male mice (Figure 2A). Although females reported an increase in aortic arch stiffness after 2 weeks, no differences were reported between 8 and 26‐week‐old mice (Male‐8 weeks: 0.47 ± 0.021 vs. 26 weeks: 0.44 ± 0.011). However, by 52‐week, aortic arch stiffness had increased in female mice (0.57 ± 0.021). When compared between genders, differences were detected at 26 weeks of age suggesting that in females the rise in aortic arch stiffness may be delayed. Assessment of ascending aorta blood flow velocity revealed that despite its rise in both males and females, there was a decline in both males and females by 26 weeks with no change after 52 weeks (Figure 2B). Changes in descending aorta blood flow velocity was limited with the only difference reported between 8 and 26‐week‐old male mice (Figure 2C). Finally, to assess whether age or body weight are important determinants, linear regression models revealed that body weight and age both influenced aortic arch stiffness (Figure 2D–G) in both male and female mice (*p* < 0.05). Interestingly, goodness of fit modeling suggests that age (*R* ^2^: 0.58) and body weight (*R* ^2^: 0.59) were greater predictors for aortic arch stiffness in female mice compared to males (age—*R* ^2^: 0.37 and body weight—*R* ^2^: 0.37).

### 3.3 LV Function Is Modestly Impacted During Early Adolescence

**FIGURE 3:**
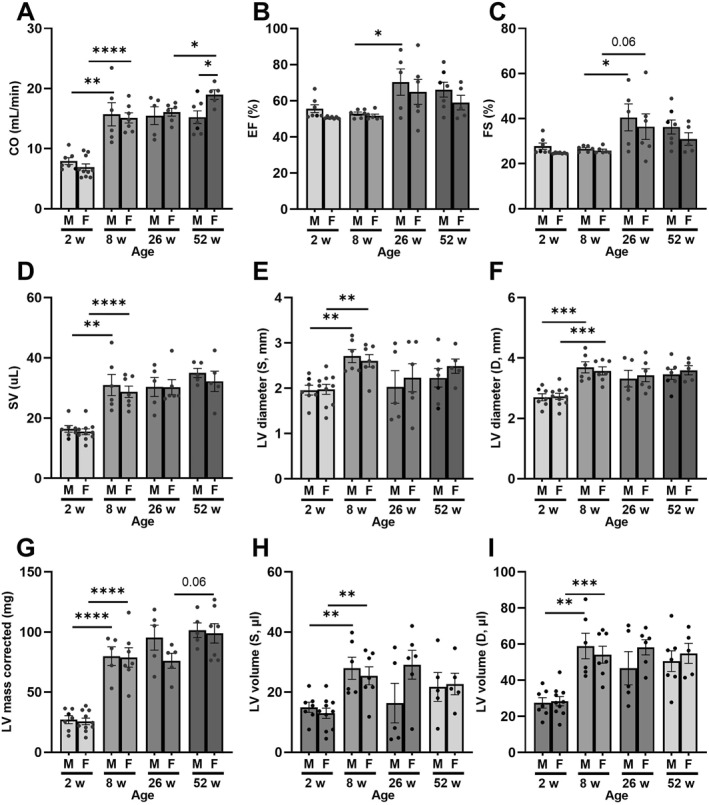
Left ventricular (LV) function of aged male and female mice assessed by echocardiography. (A) Cardiac output (CO), (B) ejection fraction (EF), (C) fractional shortening (FS), (D) stroke volume (SV), (E) LV internal diameter during diastole or (F) systole, (G) LV mass (corrected), and (H) LV volume during systole or (I) diastole. Sample size was 5–7 per group, data presented as mean ± SEM. Statistical significance presented as * *p* < 0.05, ** *p* < 0.01, *** *p* < 0.001, and **** *p* < 0.0001.

LV systolic function reported that age had a limited but noticeable impact. Irrelevant of gender, there was an increase in CO, SV, LV diameter/volume during systole and diastole, and LV mass once mice reached 2 months of age (Figure 3A,D–I). This gradual rise eventually plateaued off for almost every parameter besides EF and FS (Figure 3B,C) which only increased after 8 weeks in male but not female mice. Interestingly, CO continued to rise in 52‐week‐old female mice (compared to 26‐week‐old females) and was significantly greater compared to 52‐week‐old female mice.

**FIGURE 4:**
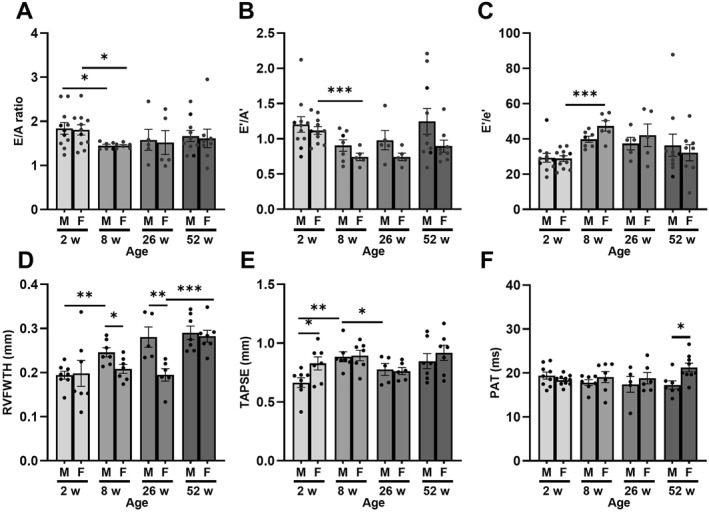
Left ventricular diastolic function and right ventricular function of aged male and female mice assessed by echocardiography. E (E) and A (A) waves in conjunction with e prime (E′) and a prime (A′) were used to determine (A) E/A, (B) ${\text{E}}^{\prime }/{\text{A}}^{\prime }$, (C) ${\text{E}}^{\prime }/{\text{e}}^{\prime }$ ratios, (D) right ventricular free wall thickness (RVFWT), (E) tricuspid annular plane systolic excursion (TAPSE), and (F) pulmonary acceleration time (PAT). Sample size was 5–10 per group, data presented as mean ± SEM. Statistical significance presented as * *p* < 0.05, ** *p* < 0.01, and *** *p* < 0.001.

Changes in LV diastolic function were limited but noticeable during the early stages of development (Figure 4). In male mice, E/A ratio was the only diastolic measurement which changed as E/A declined by 8 weeks with no further changes as they aged (Figure 4A). On the other hand, a decline in E/A and E′/A′ was reported in female mice while E′/e′ increased from 2–8 weeks of age (Figure 4A–C).

### 3.4 RV Free Wall Thickness Increases With Age With Limited Changes in RV Function

To determine whether there was an association between changes in lung function and right heart function, PAT, TAPSE, and RVFWT were assessed (Figure 4D,E,F). Our findings observed that RVFWT progressively increased in male mice from 2 to 26 weeks of age (Figure 4D). Despite this increase, the only RV functional change detected in males was the increase in TAPSE within 2–8 weeks of age (Figure 4E). Additionally, PAT did not change throughout the study suggesting that the effects of aging in middle aged male mice regarding RV function is limited (Figure 4F). In females, RVFWT also increased; however, this difference was only detected in 1‐year‐old mice while no differences were observed in the PAT or TAPSE functions. Despite this lack of differences, it is important to note that differences between males and females for PAT (at 1 year), TAPSE (2 weeks), and RVFWT (2 months) were detected. This suggests minor but significant sexual dimorphic changes in RV function occur as mice progressively age.

### 3.5 Aortic Arch Stiffness Is Moderately Correlated to the Progressive Decline in Lung Function as Mice Age

**FIGURE 5:**
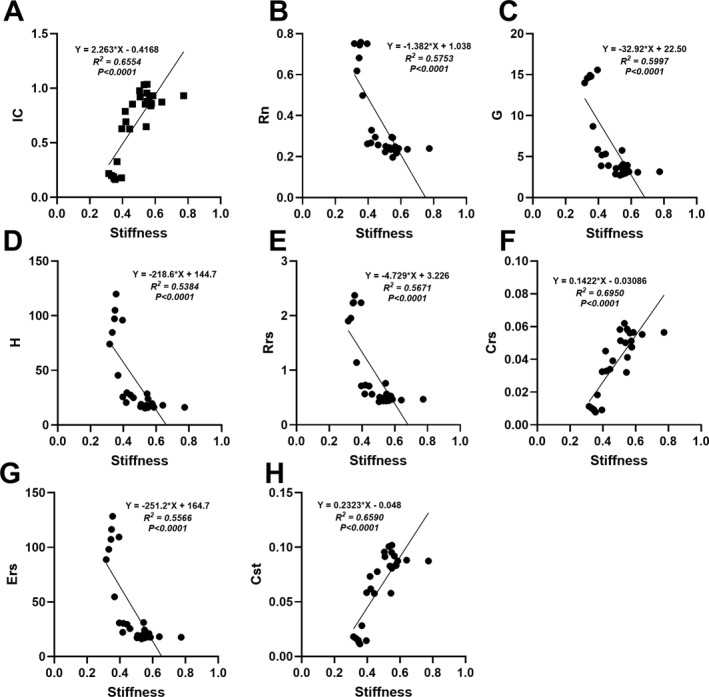
Assessment of the relationship between lung function and aortic arch stiffness in male mice. (A) Inspiratory capacity (IC), (B) Newtonian resistance (Rn), (C) tissue damping (G), (D) tissue elastance (H), (E) resistance to respiratory system (Rrs), (F) compliance of the respiratory system (Crs), (G) elastance of the respiratory system (Ers), and (H) quasi‐static compliance (Cst). Sample size was 5–16 per group.

**FIGURE 6:**
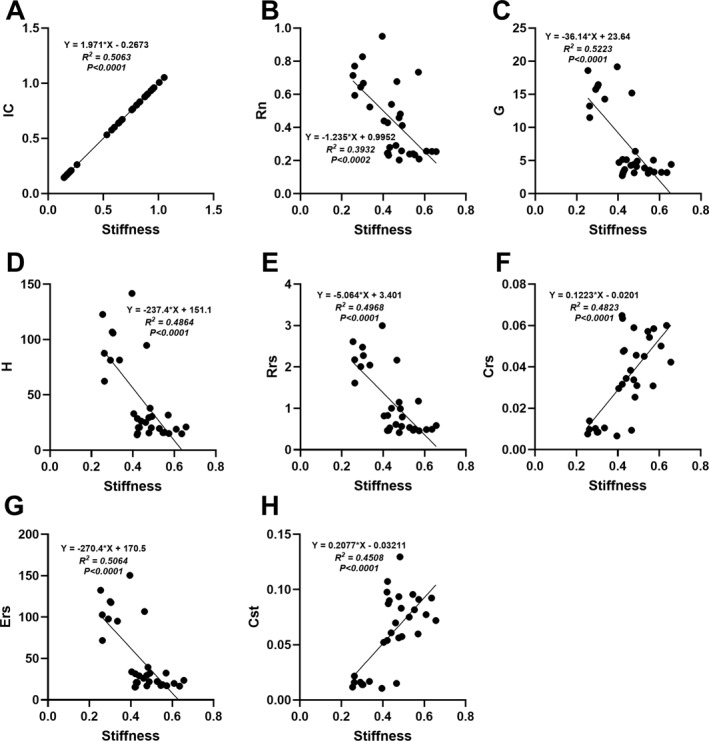
Assessment of the relationship between lung function and aortic arch stiffness in female mice. (A) Inspiratory capacity (IC), (B) Newtonian resistance (Rn), (C) tissue damping (G), (D) tissue elastance (H), (E) resistance to respiratory system (Rrs), (F) compliance of the respiratory system (Crs), (G) elastance of the respiratory system (Ers), and (H) quasi‐static compliance (Cst). Sample size was 5–16 per group.

To determine whether there was a relationship between the increasing stiffness of the aortic arch and the progressive decline in lung function, linear regression models were generated for both sexes (Figures 5 and 6). For all the lung parameters assessed, aortic arch stiffness was reported to be an important predictor in both male and female mice (*p* < 0.0001). Independent of age, positive correlations were reported for IC, Crs, and Cst (Figures 5A,F,H and 6A,F,H) whereas negative correlations were observed for Rn, G, H, Rrs, and Ers in both males and females (Figures 5B–E,G and 6B–E,G). When compared against each other, stiffness was a stronger predictor for every parametric flexiVent measurement in males (*R* ^2^: 0.53∼0.69) versus females (*R* ^2^: 0.39∼0.52).

Because of our interest in aging and its relationship with aortic arch stiffness and lung function, multiple linear regression models for these three parameters were generated (Table 1). In males, aortic arch stiffness was positively associated with the rise in IC, Crs, and Cst whereas the decline in Rn, G, H, Rrs, and Ers was negatively associated with aortic arch stiffness. Although the same trends were observed in females, statistical significance was only detected for G, H, Ers, Cst, and Rrs. As an independent variable, age was positively associated with the rise in IC, Crs, and Cst in males, whereas in females, IC and Crs were detected. Additionally, the decline in Rn and Rrs was negatively associated with aging in females but not males. Again, when compared between genders, males had slightly better goodness of fit compared to females across every parameter flexiVent measurement assessed in the multiple linear regression models.

**TABLE 1:** Multiple linear regression models investigating the relationship of age and aortic arch stiffness in multiple lung function parameters.

Male						Female			
Lung function	Variables	Estimate	Standard error	*p* value	*R* ^2^	Estimate	Standard error	*p* value	*R* ^2^
IC	Age	0.0060	0.0022	0.0055**	0.75	0.0091	0.0027	0.0019**	0.63
	Aortic arch stiffness	1.48	0.3800	0.0008****		0.62	0.46	0.18	
Rn	Age	−0.0030	0.0022	0.080	0.63	−0.0073	0.0019	0.0007***	0.61
	Aortic arch stiffness	−1.0060	0.300	0.0031**		−0.29	0.34	0.40	
G	Age	−0.0710	0.035	0.054	0.66	−0.068	0.052	0.20	0.55
	Aortic arch stiffness	−23.61	6.84	0.0021**		−27.36	9.29	0.0066**	
H	Age	−0.48	0.27	0.090	0.59	−0.55	0.36	0.14	0.53
	Aortic arch stiffness	−156.40	52.44	0.0065**		−165.80	64.85	0.017 *	
Rrs	Age	−0.0098	0.0055	0.087	0.62	−0.016	0.0073	0.036*	0.57
	Aortic arch stiffness	−3.45	1.07	0.0036**		−3.0020	1.30	0.029*	
Crs	Age	0.00044	0.0001	0.0002**	0.83	0.00056	0.00016	0.0021**	0.64
	Aortic arch stiffness	0.085	0.019	0.0002**		0.050	0.029	0.10	
Ers	Age	−0.53	0.30	0.093	0.61	−0.56	0.40	0.17	0.54
	Aortic arch stiffness	−182.80	58.16	0.0044**		−197.60	71.38	0.01*	
Cst	Age	0.00064	0.00020	0.0035**	0.76	0.00058	0.00034	0.094	0.51
	Aortic arch stiffness	0.15	0.038	0.0007***		0.13	0.060	0.037*	

*Note:* Sample size was 5–16. Statistical significance presented as *p < 0.05, **p < 0.01, ***p < 0.001 and ****p < 0.0001.

Abbreviations: Crs, compliance to respiratory system; Cst, quasi‐static compliance; Ers, elastance of the respiratory system; G, tissue damping; H, tissue elastance; IC, inspiratory capacity; Rn, Newtonian resistance; and Rrs, resistance to respiratory system.

## 4 Discussion

The inverse relationship between the decline in lung function and aging is a well‐established phenomenon which is attributed to structural changes of the respiratory system [10]. Increased lung stiffness and reduced elasticity cause a decline in respiratory inspiratory/expiratory capacity. Consequently, this increases cardiac workload as the heart must compensate for the decline in pulmonary function which increases the risk of developing cardiac hypertrophy and heart failure. Despite their close relationship, there are no early markers or methods to track changes in cardiopulmonary function. In the last few decades, aortic arch stiffness has been recognized as an important parameter of cardiovascular risk assessment [8, 9]. The aim of this study was to determine whether the stiffness of the aortic arch was correlated to changes in lung function in aged male and female mice.

Our results revealed conflicting findings regarding the change in lung function as mice aged. Despite the increase in inspiratory capacity and lack of stiffness detected in the lungs, lung elasticity significantly declined in 1 year old mice. Interestingly, most of these changes occur during the early stages of adolescence with limited to no changes in lung function after 2 months. The rapid changes in lung function from 2 to 8 weeks of age were of particular interest and highlighted the significant developmental changes which occur from adolescence to adulthood [11, 12, 13]. Alveoli development, the change to collagen‐to‐elastin ratio, and remodeling of the pulmonary extracellular matrix are all known to be involved in these changes. The rapid decline in tissue elasticity has been previously reported during adolescence and suggests that this is partly due to increased levels of collagen which leads to the decline in lung elasticity [11]. However, as reported by our study, this does not affect inspiration capacity or lung compliance. Our study also observed that after 1 year, no decline in respiratory function was detected in aged mice. These findings are supported by previous aging studies [10, 14] which reported similar findings and observed that inspiratory capacity only begins to decline after 2 years. Considering that the average lifespan of C57 mice is approximately 3 years [15], lung function in mice appears to only decline noticeably after 2 years. In similar studies [10, 16] which have investigated the impact of aging, researchers have shown that aged mice (greater than 2 years) have increased alveolar size, enlargement of alveolar ducts as well as increased lung volume. Additionally, the decline in lung elasticity due to reduced collagen‐to‐elastin consequently impacts expiratory capacity as the ability to recoil and expel air efficiently is reduced. However, considering that these findings are only observed in old mice, the assessment of age in only adolescent–middle‐aged mice may be a poor predictor for pulmonary function as its decline is nonlinear and remains consistent throughout most of their lifespan. It is also important to consider that this study does not examine environmental (pollution or socio‐economic) factors or lifestyle (smoking or exercise) choices which likely affect the rate at which lung function declines as we age [4].

Despite these discrepancies, cardiac function had similar trends as LV and RV function increased during maturation with no significant changes observed once they reached middle age. These findings reflect the fact that cardiac workload increases as the heart grows during early development before plateauing off once maturity is reached around 3–6 months. Additionally, myocardial function only begins to decline by 24 months which is considered biologically “old” for C57BL/6J mice [17, 18, 19]. Despite this, the rise in aortic arch stiffness does suggest a progressive stiffening of the aortic arch which is correlated with the change in pulmonary function and age. These findings are supported by multiple clinical studies which have reported aortic arch stiffness to be positively associated with reduced lung function, especially in aging populations [20, 21, 22]. In the context of aging, the increase of aortic arch stiffness is not only directly correlated to the increased levels of collagen and elastin but also to its uneven distribution [23, 24, 25]. It would be of interest to determine whether the correlation between aortic arch stiffness and lung function strengthens as mice progressively age from 2 to 3 years. This is especially important when considering the effects of gender. Although our study reported limited differences between males and females, it is interesting to note that for every parameter of lung function assessed in this study, the correlation among age, aortic arch stiffness, and lung function was greater in males compared to females. These findings reflect the sexual dimorphic differences in lung/heart size, anatomy, and structure which are known to differ between genders [26]. Endocrine is also another important factor which has been reported to influence the cardiopulmonary system. Estrogen is known to have anti‐inflammatory properties in females and has been previously reported to be a protective factor in the cardiopulmonary system [27, 28, 29]. Interestingly, this protective phenomenon is lost in postmenopausal females suggesting that age is a significant factor in influencing the cardiopulmonary system. In the context of this study, the limited changes observed in females may be attributed to the fact that only mice up to 1 year of age were used. Although the reproductive senescence of mice occurs between 9 and 12 months of age, the number of females which transition to a menopausal state which, comparable to humans, is limited [30]. The use of rodent models to recapitulate the phenotype (genetically modified animals, chemical induction, and ovariectomy) observed in human menopause could be used in future studies to investigate the relationship between aortic arch stiffness and pulmonary function in the context of aging.

There are several limitations in this study. Firstly, this study only examined mice aging from 2 to 52 weeks and does not adequately reflect changes in cardiopulmonary function in older mice. Assessing cardiopulmonary function in older C57 mice of at least 2 years of age would offer a more accurate understanding of the effects of aging. Furthermore, our study did not explore why there was a decline in lung elasticity at the molecular level. Previous studies have demonstrated that the decrease in lung elasticity is attributed to the loss of elastin and collagen fibers, as well as tissue remodeling that occurs over time [31, 32, 33]. It would be interesting to assess the development of the extracellular matrix during the transition from adolescence to adulthood since most of the changes observed during this study were from 2 to 8 weeks of age. Another target is Werner's syndrome protein which has also been suggested to correlate with lung function during aging [34]. Future studies involving the use of older mice in our study and examining the level/distribution of elastin, collagen, and Werner's syndrome protein would warrant further investigation. Finally, although this study focused on investigating the effects of aging independent of environmental or lifestyle factors, our findings are still relevant and highlight the relationship between aortic arch stiffness and lung function.

## 5 Conclusion

Our findings suggest that an inverse correlation between the increase in aortic arch stiffness and the decline in lung function develops in mice as they progressively age. This correlation could serve as an early predictor for evaluating the decline in cardiopulmonary function. These findings highlight the potential of aortic arch stiffness as an early indicator of aging‐related cardiopulmonary decline and warrant further exploration.

## Author Contributions

A.I., J.C., S.M.L., and I.L. conceptualized the study. S.M.L., J.C., and A.I. drafted the manuscript, designed and prepared the figures. A.I. performed all the mouse lung function measurements and data analysis; J.C. and S.M.L. performed mouse echocardiographic imaging; S.M.L., A.I.S., S.C., and D.A.M. provided mouse echocardiographic data analysis; Y.Y performed covariant analysis (Table S1); I.L., M.C., R.D.M., and J.T. provided resources and technical support. The manuscript was revised by S.M.L., A.I., and J.C. All the authors have reviewed and approved the final version of the manuscript for publication.

## Ethics Statement

The in vivo experiments from this study were approved by University of Illinois at Chicago's Institutional Animal Care and Use Committee, approval number 23‐073.

## Conflicts of Interest

The authors declare no conflicts of interest.

## Supporting information

Supporting Information S1

## Data Availability

The data that support the findings of this study are available from the corresponding author upon reasonable request.
