# Supplementary material for: Age‐Dependent Correlation of Pulmonary Function Impairment and Aortic Arch Stiffness in Mice
Source: Pediatr Discov. 2025 Apr 2;4(1):e2525. doi: 10.1002/pdi3.2525 (PMC13098100; doi:10.1002/pdi3.2525)
Supplement: Supplementary file 1 — Supporting Information S1 [file PDI3-4-e2525-s001.docx]

**Supplementary Figures and Tables**

**Supplementary Figure 1.**

**Figure S1**. Assessment of the relationship between lung function and age in male mice. **A** - Inspiratory capacity (IC), **B** - Newtonian resistance (Rn), **C** – Tissue dampening (G), **D** – Tissue elastance (H), **E** – Resistance to respiratory system (Rrs), **F** - Compliance to respiratory system (Crs), **G** – Elastance of the respiratory system (Ers), **H** – Quasi-static compliance (Cst). Sample is 5-16 per group.

**Supplementary Figure 2.**

**Figure S2**. Assessment of the relationship between lung function and body weight in male mice. **A** - Inspiratory capacity (IC), **B** - Newtonian resistance (Rn), **C** – Tissue dampening (G), **D** – Tissue elastance (H), **E** – Resistance to respiratory system (Rrs), **F** - Compliance to respiratory system (Crs), **G** – Elastance of the respiratory system (Ers), **H** – Quasi-static compliance (Cst). Sample is 5-16 per group.

**Supplementary Figure 3.**

**Figure S3**. Assessment of the relationship between lung function and age in female mice. **A** - Inspiratory capacity (IC), **B** - Newtonian resistance (Rn), **C** – Tissue dampening (G), **D** – Tissue elastance (H), **E** – Resistance to respiratory system (Rrs), **F** - Compliance to respiratory system (Crs), **G** – Elastance of the respiratory system (Ers), **H** – Quasi-static compliance (Cst). Sample is 5-16 per group.

**Supplementary Figure 4.**

Figure S4: Assessment of the relationship between lung function and body weight in female mice. **A** - Inspiratory capacity (IC), **B** - Newtonian resistance (Rn), **C** – Tissue dampening (G), **D** – Tissue elastance (H), **E** – Resistance to respiratory system (Rrs), **F** - Compliance to respiratory system (Crs), **G** – Elastance of the respiratory system (Ers), **H** – Quasi-static compliance (Cst). Sample is 5-16 per group.

**Supplementary Table 1.**

**
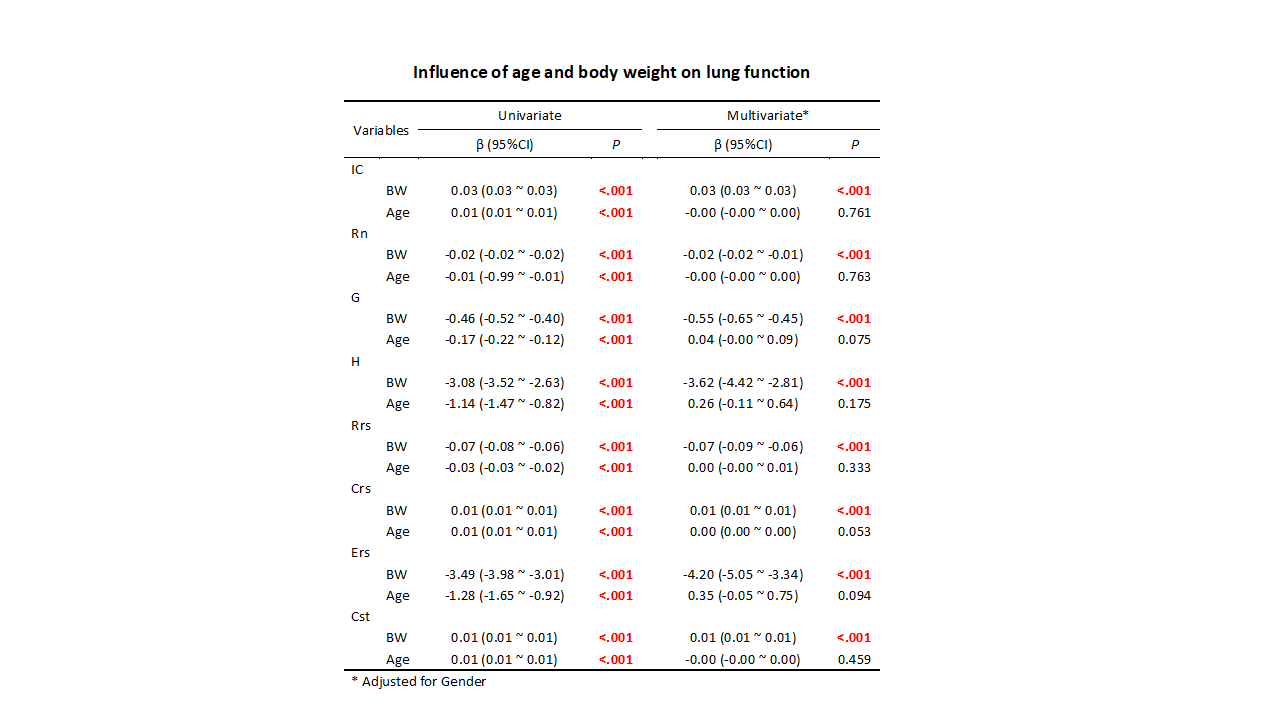
**

**Table S1**. ANCOVA univariate / multivariate analyses examining the correlation of age and body weight (BW) with Inspiratory capacity (IC), Newtonian resistance (Rn), Tissue dampening (G), Tissue elastance, Resistance to respiratory system (Rrs), Compliance to respiratory system, Elastance of the respiratory system (Ers) and Quasi-static compliance (Cst).
